# Comparing machine learning, deep learning, and reinforcement learning performance in *Culex pipiens* predictive modeling

**DOI:** 10.1371/journal.pone.0333536

**Published:** 2025-11-13

**Authors:** Wei Yin, Sanad H. Ragab, Michael G. Tyshenko, Teresa Feria Arroyo, Tamer Oraby

**Affiliations:** 1 School of Mathematical and Statistical Sciences, The University of Texas Rio Grande Valley, Edinburg, Texas, United States of America; 2 Department of Zoology and Entomology, Faculty of Science (Boys), Al-Azhar University, Nasr City, Cairo, Egypt; 3 Risk Sciences International, Ottawa, Canada; 4 School of Integrative Biological and Chemical Sciences, The University of Texas Rio Grande Valley, Edinburg, Texas, United States of America; National Research Centre, EGYPT

## Abstract

Several machine learning (ML) and deep learning (DL) methods have been used to predict the presence of species in classification problems. Another set of methods, called reinforcement learning (RL), has been used in training agents to perform various tasks, but not in predicting species distribution. *Culex pipiens* (Diptera: Culicidae), commonly known as the common house mosquito, is a globally distributed species prevalent in temperate and subtropical regions. They serve as a primary vector for West Nile Virus (WNV), a mosquito-borne pathogen that affects humans and other animals. The study objective is to compare the performance of logistic regression, random forest classifier, deep neural networks, and the RL methods, including Q-learning, deep Q-network (DQN), REINFORCE, and Actor-Critic, in predicting the historical presence of *C. pipiens* through their potential geographic distribution in the USA. The comparison showed similar performance across approaches, with reinforcement learning methods like DQN and REINFORCE showing effective performance using fewer features, making them as great prediction tools for changing environments or situations with limited resources. Moreover, the results revealed that altitude and annual precipitation were the most important bioclimatic variables predicting the historical presence of *C. pipiens*.

## Introduction

Accurately predicting mosquito distribution is essential for effectively managing and controlling mosquito-borne diseases (MBD). Several machine learning techniques can be employed to address such complex ecological challenges. Logistic Regression [1] and Random Forest [2] are well-suited methods for classification. Logistic Regression is particularly useful for binary classification problems, such as predicting the presence or absence of mosquitoes with climatic variables as inputs. The Random Forest (RF) excels at handling nonlinearity of variables and complex interactions between them. RF is very capable of classification and regression tasks in ecological studies. Besides, the Maximum Entropy Model (MaxEnt) is widely used in ecological and environmental sciences for modeling species distributions [3]. It can make predictions as uniform as possible while still fitting the observed data. This characteristic makes the MaxEnt model well-suited for real-world applications where information about the absence of target species is difficult to obtain.

The Deep Neural Network (DNN) method is another robust approach used in pattern recognition and regression. DNNs, with their multiple layers and non-linear activation functions, can model intricate relationships within data and handle high-dimensional inputs. They effectively capture complex patterns and interactions in large datasets, which can assist in predicting future trends in mosquito distribution.

Reinforcement Learning (RL) [4] is a more advanced approach that incorporates several sophisticated techniques, distinct from traditional supervised and unsupervised machine learning methods. RL focuses on training and enabling one or more agents to make sequential decisions to maximize cumulative rewards within an environment. In particular, the agent can utilize various RL algorithms, such as Q-learning, Deep Q-Network (DQN), and policy gradient methods, including REINFORCE and Actor-Critic (AC), to learn a policy that maps states to actions in a way that maximizes cumulative rewards. In the context of mosquito distribution, the agent learns to predict mosquito distribution by identifying patterns in environmental data associated with their historical presence. Moreover, the model would iteratively improve its predictions as it learns from the data and updates its policy.

Mosquitoes are the main vectors of several pathogens that cause human diseases such as West Nile encephalitis, Rift Valley Fever, and Lymphatic filariasis [5–7]. Among these, *C.pipiens* complex mosquitoes are major vectors of pathogens worldwide. Although native to Africa, *C.pipiens* complex mosquitoes have spread to many regions around the world. In North America, *C.pipiens* is found in the northern United States and southern Canada, in areas above $39^{\circ }$ north latitude [8]. In contrast, the closely related *Culex quinquefasciatus*, known as the southern house mosquito, is found below $36^{\circ }$ north latitude. Between $32^{\circ }$ and $40^{\circ }$ north latitudes, there is a broad hybridization zone where *C. pipiens*, *C. quinquefasciatus*, and their hybrids co-exist [9].

Environmental conditions are important determinants of the distribution and reproduction of *C.pipiens* complex mosquitoes [9–11]. Furthermore, temperature affects the maturation and replication rates of pathogens within mosquitoes, increasing the likelihood of infection [12,13]. For diseases transmitted by vectors that have aquatic developmental stages, such as mosquitoes, precipitation plays a crucial role in the dynamics of these diseases, influencing them in various ways depending on the ecological characteristics of the mosquito vectors [14]. Notably, the incidence of mosquito-borne diseases has been observed to increase in response to climate change. Over the last 20 years, the prevalence of mosquito-borne diseases in Canada has risen by 10%, an increase largely attributed to climate change [15].

We were motivated to investigate the use of RL as it has several advantages when used for modeling mosquito populations in dynamic environments. Mosquito populations can be influenced by multiple interacting ecological and climatic variables. Unlike traditional supervised learning models, RL agents can learn to apply optimal policies through feedback loops, enabling them to adapt to changing environmental conditions such as precipitation, temperature, and altitude. The adaptability of the RL algorithm reduces the potential for overfitting that is often seen with static data [16]. Moreover, it allows the model to generalize more effectively over time. RL performs well for decision-making under uncertainty, making it particularly suitable for real-time entomological surveillance data where environmental inputs and mosquito population dynamics can change rapidly. In addition, RL can operate with infrequent, sporadic, sparse, or delayed reward signals, which is helpful if surveillance data on mosquito populations are limited or noisy. These features make RL a superior choice for predictive modeling in applications such as WNV mosquito transmission, public health vector management, and ecological forecasting.

In this paper, we compare the performance of Machine Learning, Deep Learning, and Reinforcement Learning algorithms in predicting the presence of *C. pipiens*. Predictive modeling has been widely used to explore human disease outcomes [17,18]; however, very few studies have systematically compared these various algorithmic approaches in the context of insect disease transmission and surveillance. To our knowledge, this is the first study to apply AI methods (ML, DL, and RL) comparatively specifically for vector surveillance and early prediction of disease vector hotspots.

Historical data of the presence of the *C. pipiens* mosquito, and corresponding bioclimatic conditions were used to train these models. Since those ML, DL, and RL methods were applied for classification, the primary goal was to compare their performance. We used randomly sampled absences predicted by MaxEnt trained on the same presence data and published in [19]. We considered the absences produced by MaxEnt to be ground truth in this study. The models were validated using a separate dataset to ensure their accuracy in predicting the presence of mosquitoes. The trained models, which account for the complex interactions between environmental factors and mosquito presence, were used to make predictions across the USA.

## Materials and methods

### Mosquito data

The occurrence data of *C. pipiens* was obtained from the Global Biodiversity Information Facility (GBIF.org, https://doi.org/10.15468/dl.sgpgg0) on 7 April 2023. The downloaded database hosts 1315 geo-referenced records (1950–2018), including coordinates. The source of these occurrence data is human observations and preserved specimens. Those records were verified using ArcGIS 10.3 ESRI [20]. Records outside the base map (USA) shapefile (ESRI) or located in water were removed from the analysis. Additionally, duplicate geographical records were removed. This resulted in 1,313 presence points, which were further reduced to 1,308 records after deleting the reciprocated missing values of the resampled bioclimatic variables of climate and topography. The MaxEnt model [19] was also employed to produce 1308 absence data that were randomly selected from the map. The final dataset comprises 2614 observations, which are balanced between presence and absence. The maximum area under the curve (AUC) in [19] was found to be .77. That said, the performance of the methods here is constrained in predicting the presence of *C. pipiens* by the predictive performance of the MaxEnt method as in [19].

### Environmental data

Twenty variables were selected as predictors to model the potential environmental niche of *C. pipiens* based on its current presence data. Specifically, 19 bioclimatic layers (bio01–bio19) and a topographic variable (elevation) were obtained from the WorldClim database (http://www.worldclim.org/) at a spatial resolution of 2.5 arc-minutes (5 km × 5 km at the equator) [21]. These variables were selected based on previous studies that identified them as the most significant factors for modeling potential species distribution [22].

### Data preprocessing

Mosquito occurrence data and environmental data were combined into a single structured dataset. Before modeling, the data were normalized using min-max scaling to ensure that all features contributed equally to the model performance. In this study, all statistical analyzes were conducted using Python. The optimal hyperparameters for all models were determined through the Grid Search method combined with 5-fold cross-validation. To evaluate the processes, we adopted an 80:20 train-test random data split. Model performance metrics were evaluated on the testing dataset. In addition, the four RL methods were trained using 10 different random seeds, and their average performance was calculated.

### Pattern visualization and multivariate analysis

We began by performing a correlation analysis among all features, creating a scatter matrix plot to visualize the relationships between variables, and calculating the Variance Inflation Factor (VIF) to identify potential multicollinearity. VIF values were computed by regressing each feature against all other features and calculating the factor using the formula: $VIF=\frac{1}{1-R^{2}}$, where *R*^2^ is the coefficient of determination. VIF of more than 5 indicates the presence of multicollinearity between the features. Since multicollinearity reduces the reliability of estimates for feature effects in a model, it is necessary to address it by eliminating some features.

### Feature extraction

We applied Principal Component Analysis (PCA) as a feature extraction technique to reduce the dataset’s dimensionality. By retaining the principal components that explain a significant portion of the variance, typically 95% or 99%, PCA effectively summarizes the data while preserving most of its informational content. PCA works by transforming the original features into a new set of uncorrelated variables, known as principal components. In addition to PCA, SHapley Additive exPlanations (SHAP) was implemented to provide information on the contribution of each feature to the model’s prediction. A permutation test was also applied to determine whether a feature is significant using the simulated p-value.

### Machine learning models

At first, we employed Logistic Regression to model the relationship between the binary dependent variable (presence or absence of *C. pipiens*) and multiple features by estimating the probabilities associated with the binary outcome variable [23]. Random Forest is another machine learning algorithm for classification problems. Unlike traditional regression models, RF does not rely on individual regression formulas; instead, it constructs multiple decision trees and combines their results to produce a more accurate and stable prediction [24]. After parameter tuning, the Random Forest classifier was configured with 200 trees, a maximum depth of 20, 15 maximum features, a minimum sample per leaf of 1, and a minimum samples per split of 2. For more information, see S1 Appendix.

### Deep learning models

Deep learning is a machine learning method that focuses on utilizing neural networks to perform tasks such as classification, regression, and representation learning. We implemented the DNN model to learn and represent complex patterns in the data [25]. A DNN is an artificial neural network (ANN) with multiple layers between the input and output layers [26]. The components of DNN—such as neurons, weights, biases, and functions—work together like a simplified version of the human brain and can be trained like any other machine learning algorithm. In this study, the DNN model is implemented using the Keras Sequential API. It consists of a feedforward multilayer perceptron designed for binary classification. The model includes an input layer that accepts all standardized features, followed by three hidden layers, each with 64 ReLU-activated neurons and L2 regularization to reduce overfitting. Batch normalization is applied after each dense layer to stabilize and accelerate training. Additionally, dropout layers with a 0.2 rate are included after the second and third hidden layers to enhance generalization. The output layer consists of a single neuron with a sigmoid activation function, which produces probabilistic outputs for binary predictions. The model is compiled using the Adam optimizer with a learning rate of 0.001 and the binary cross-entropy loss function. This structure enables the model to effectively identify complex patterns within the dataset. However, the inherent complexity of DNNs reduces their interpretability, making it challenging to understand the exact reasoning behind their predictions.

### Reinforcement learning models

Reinforcement learning is an interdisciplinary area of machine learning and optimal control that focuses on how agents can make sequences of decisions to maximize cumulative rewards in an environment [4]. In RL, an agent interacts with an environment and learns to make optimal decisions through trial and error. The core components of a reinforcement learning problem are the agent, which is the learner or decision-maker; the environment, which represents everything external to the agent; and the reward, a scalar value that guides the agent by indicating the desirability of particular actions and/or states.

The learning process in RL is characterized by a feedback loop. The agent observes the current state of the environment, takes action based on its policy, and receives feedback in the form of a reward and a new state. Over time, the agent adjusts its policy to favor actions that lead to higher cumulative rewards. This adjustment is usually achieved using methods such as value functions, which estimate expected rewards for states or actions, or policy optimization, which directly adjusts the agent’s strategy.

In this paper, the environment represents geographic and ecological regions where *C. pipiens* mosquitoes could potentially inhabit. These regions are characterized by climate variables, including temperature, humidity, and precipitation, as well as geographic factors such as elevation, altitude, and longitude. The state is designed to capture different environmental conditions, represented as a vector of several climate variables such as temperature, humidity, and precipitation levels in a specific geographic area. The agent’s action is binary, with two possible values: zero, indicating the absence of *C. pipiens*, and one, indicating their presence.

In addition, four main sub-elements comprise a reinforcement learning system: a policy, a reward, a value function, and, optionally, an environment model. They are described in more detail:

A **policy** defines how a learning agent behaves at a particular time by mapping perceived states of the environment to actions to be taken in those states.

A **reward** defines the goal in a reinforcement learning problem. At each time step, the environment sends the reward to the agent. The agent’s objective is to maximize its total reward over training time. The reward an agent receives depends on its current action and the state of the environment, and the agent cannot modify this process.

A **value** function estimates how good it is for the agent to be in a given state (or how good it is to perform a given action in a given state). More specifically, the value of a state is the total amount of reward an agent can expect to accumulate in the future, starting from that state.

A **model** of an environment refers to a mechanism that simulates the behaviors of the environment, allowing inferences about its future actions. In this study, the learning approaches are based on trial-and-error experience, making it a model-free method.

We employ several RL algorithms and compare their performance with that of the ML and DL methods, as discussed below.

#### Q-Learning.

Q-Learning is a type of model-free reinforcement learning algorithm that makes optimal decisions in an environment [27]. The state-action quality function, denoted by *Q*, is defined as the expected cumulative reward that an agent can achieve by starting in a particular state, taking a specific action, and following the optimal policy thereafter. The Q-Learning algorithm iteratively updates the *Q* values for state-action pairs using the following Bellman equation [28,29]:

$$Q(s,a^{*})=\max_{a}\left(R(s,a)+\gamma \sum_{s^{\prime }}P(s^{\prime }|s,a)Q(s^{\prime },a^{\prime })\right)$$ (1)

where *Q*(*s*, *a*) is the *Q* function for state-action pair (*s*, *a*); *R*(*s*, *a*) is the immediate reward for taking action *a* in state *s*; *γ* is the discount factor, such that γ∈[0,1), which characterizes how the utility of rewards decays based on how far in the future they occur. Finally, $P(s^{\prime }|s,a)$ is the transition probability from state *s* to state $s^{\prime }$ when taking action *a*.

While running the Q-learning algorithm, the agent will encounter various options and select different actions. To find the best action, the agent tabulates all the findings in a table called a Q-Table. Bellman equation (1) at each state is applied to get the expected future state and reward that is saved in the Q-Table to compare to other states. This is how the Q-Table assists in finding the best action for each state in the environment.

In all RL models applied in this study, the state space was defined by the selected bioclimatic features. In contrast, the action space corresponded to the binary classification outcomes of the presence or absence of *C. pipiens*. Furthermore, all RL models have been trained for over 2000 episodes and evaluated on more than 1000 testing episodes. The Q-Learning model was trained using a learning rate of 0.3, allowing for fast updates to the Q values, and a discount factor of 0.8, balancing the immediate and future rewards. An exploration probability of 0.1 is used to ensure sufficient exploration of the state action space.

#### Deep Q Network (DQN).

Deep Q Network is another reinforcement learning algorithm utilized in this paper [30]. The idea behind DQN is to implement deep neural networks to estimate the value function. Due to its ability to address complex and high-dimensional state-action spaces, DQN is widely applied in various fields, including healthcare, robotics, autonomous vehicles, natural language processing, and finance. The DQN model was implemented as a fully connected feedforward neural network including three dense layers. The first hidden layer consisted of 64 neurons with ReLU activation, followed by a second hidden layer with 16 neurons. The final output layer contained two neurons, corresponding to the estimated Q-values for each possible action in the action space. The model was trained using a learning rate of 0.01 and batch size of 32.

Both Q-learning and DQN are value-based methods; the algorithm learns the optimal action-state value function *Q*, and then the agent takes an action concerning the learned function *Q*.

#### REINFORCE.

REINFORCE [31], another model employed for predicting the presence of *C. pipiens*, is treated as a classic policy-based algorithm. Unlike value-based methods mentioned above, which learn a value function to derive a policy, policy-based methods involve training the agent to directly learn the optimal policy from states to actions without explicitly learning the value function [31,32]. In this approach, the policy is parameterized by a weight vector *θ*, so that changing *θ* changes the policy. The function $\pi_{\theta }(a|s)$ denotes a parameterized policy with weight vector *θ* for action *a* taken when the environment is in state *s*. The algorithm iteratively adjusts these parameters to enhance the probability of favorable actions based on a policy gradient theorem (PGT). The PGT states that for any differentiable policy $\pi_{\theta }(a|s)$, and for a policy objective function *J*, the policy gradient is


$$\nabla_{\theta }J(\theta )\propto \sum_{s}d^{\pi }(s)\sum_{a}Q^{\pi_{\theta }}(s,a)\nabla_{\theta }\pi_{\theta }(a|s)$$

Therefore, the theorem enables us to compute the gradient of the objective function with respect to the policy parameters, eliminating the need to consider the derivative of the state distribution. Following parameter tuning, the REINFORCE model has been trained using a discount factor of 0.9 and an exploration probability of 0.05.

#### Actor-critic.

Actor-Critic is another policy-based method implemented to analyze the data set [33,34]. It has a more complex architecture comprising two neural network models. The actor-network is tasked with learning and representing the policy, selecting actions based on the current state to maximize the expected cumulative reward. It takes the state as input and generates a policy, expressed as a probability distribution over actions. The Critic is designed to estimate the value of the state and take a particular action. Utilizing a similar neural network structure, the Critic takes both the state and action as input and outputs the Q-value.

In the A2C model, the actor learning rate was set at 0.01, which determines how much the actor’s policy is updated in response to feedback from the critic. The critic learning rate was 0.05, controlling how much the value function is updated when new rewards are observed. The discount actor was set to 0.98, balancing the importance of immediate and future rewards in the learning process.

### Evaluation metrics

To compare model performance, we utilized several standard classification metrics, including accuracy, precision, recall, and F1 score. These were computed based on the test dataset, which is not used during training.

## Results

### Influence of factors on Culex pipiens

**Fig 1:**
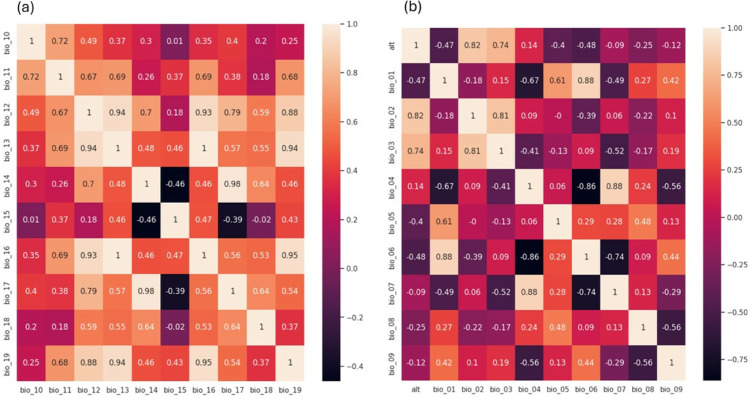
Correlation matrix. Correlation matrix illustrating the correlation coefficients between variables: (a) alt, bio_01, bio_02, bio_03, bio_04, bio_05, bio_06, bio_07, bio_08, and bio_09, and (b) bio_10, bio_11, bio_12, bio_13, bio_14, bio_15, bio_16, bio_17, bio_18, and bio_19

Correlation coefficients between the following pairs: location’s altitude (alt) and mean diurnal range (bio_02), annual mean temperature (bio_01) and minimum temperature of coldest month (bio_06), mean diurnal range (bio_02) and isothermality (bio_03), and temperature seasonality (bio_04) and temperature annual range (bio_07) are all greater than 0.8 as demonstrated in Fig 1(a). That indicates strong positive linear relationships between those pairs. Conversely, the correlation coefficients between the following pairs: temperature seasonality (bio_04) and minimum temperature of the coldest month (bio_06), and the latter variable and temperature annual range (bio_07) fall below –0.7. That suggests a strong negative linear relationship between them. Other variables, such as altitude (alt) and isothermality (bio_03) also exhibit a positive correlation, while annual mean temperature (bio_1) and temperature seasonality (bio_04) demonstrate a negative association.

Another strong positive linear association is observed among the pairs: annual precipitation (bio_12) and precipitation of wettest month (bio_13), (bio_12) and precipitation of wettest quarter (bio_16), (bio_12) and precipitation of driest quarter (bio_17), and (bio_12) and precipitation of coldest quarter (bio_19), as indicated in Fig 1(b). This suggests that these precipitation-related variables are closely related and tend to increase or decrease together. Similarly, the pairs: mean temperature of warmest quarter (bio_10) and mean temperature of coldest quarter (bio_11), mean temperature of coldest quarter (bio_11) and annual precipitation (bio_12), and annual precipitation (bio_12) and precipitation of driest month (bio_14) are observed to exhibit positive associations with each other.

High correlation among predictor variables can lead to issues in fitting and interpreting regression models. This high correlation is an indicator of multicollinearity, which can compromise the accuracy of the model.

**Fig 2:**
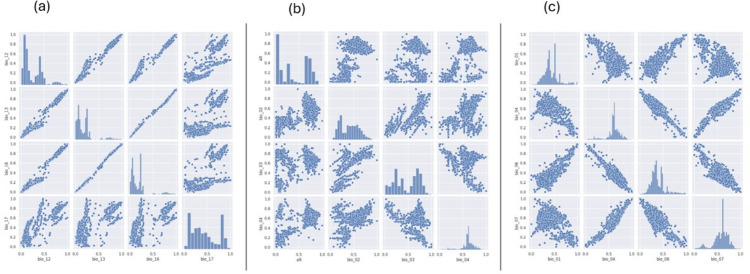
Scatter plot matrix. Scatter plot matrix illustrating the association among variables: (a) alt, bio_02, bio_03, and bio_04, (b) bio_01, bio_04, bio_06, bio_07, and (c) bio_12, bio_13, bio_16, bio_17

A positive linear relationship is observed between the variables altitude (alt) and mean diurnal range (bio_02), as well as between mean diurnal range (bio_02) and isothermality (bio_03), as shown in Fig 2(a). Meanwhile, a negative linear relationship is observed between isothermality (bio_03) and temperature seasonality (bio_04). Additional strong negative linear associations between temperature seasonality (bio_04) and min temperature of the coldest month (bio_06), as well as between temperature seasonality (bio_04) and temperature annual range (bio_07), are illustrated by Fig 2(b). Similarly, negative relationships are observed between the mean yearly temperature (bio_01) and temperature seasonality (bio_04), and between the annual mean temperature (bio_01) and the annual temperature range (bio_07).

An increase in annual precipitation (bio_12) leads to higher values of the variables of precipitation of wettest month (bio_13), precipitation of wettest quarter (bio_16), and precipitation of driest quarter (bio_17) in Fig 2(c), revealing positive linear relationships among them. These findings are all consistent with the earlier analysis.

The estimated Variance Inflation Factor (VIF) values for all bio-climatic features can be found in Table 1. The table output indicates that the majority VIF values of the variables are significantly high. Specifically, the highest value is captured for the mean temperature of the warmest quarter (bio_10), followed by temperature seasonality (bio_04), temperature annual range (bio_07), and mean temperature of the coldest quarter (bio_11). On the other hand, the mean temperature of the driest quarter (bio_09) is the variable with the lowest VIF value at 22.571. A higher VIF indicates a stronger degree of multicollinearity, suggesting that the feature may be highly correlated with other features. Consequently, the presence of such multicollinearity among these features can lead to a model with reduced stability and reliability.

**Table 1:** Estimated Variance Inflation Factor (VIF) values for 20 climatic features.

Feature–VIF		Feature–VIF		Feature–VIF		Feature–VIF	
alt	69.141	bio_01	1363.606	bio_02	595.108	bio_03	458.560
bio_04	2778.034	bio_05	1816.015	bio_06	1174.487	bio_07	2434.195
bio_08	33.411	bio_09	22.571	bio_10	5544.658	bio_11	2325.558
bio_12	953.668	bio_13	1231.087	bio_14	301.366	bio_15	23.143
bio_16	1586.096	bio_17	519.019	bio_18	48.101	bio_19	133.729

The permutation test helps determine whether the observed relationship is statistically significant by repeatedly permuting the feature values and measuring the effect on the model’s performance. The results of the permutation tests are summarized in Table 2. Among all features, only the precipitation of the driest month (bio_14) has a p-value greater than 0.05, indicating that most features, except the precipitation of the driest month (bio_14), exhibit a statistically significant nonrandom relationship with the target variable.

**Table 2:** Significance of features.

Feature–P-value		Feature–P-value	
alt	0.0000	bio_10	0.0000
bio_01	0.0000	bio_11	0.0000
bio_02	0.0200	bio_12	0.0000
bio_03	0.0000	bio_13	0.0000
bio_04	0.0000	bio_14	0.1120
bio_05	0.0000	bio_15	0.0000
bio_06	0.0000	bio_16	0.0000
bio_07	0.0060	bio_17	0.0230
bio_08	0.0000	bio_18	0.0000
bio_09	0.0000	bio_19	0.0000

### Feature importance

**Fig 3:**
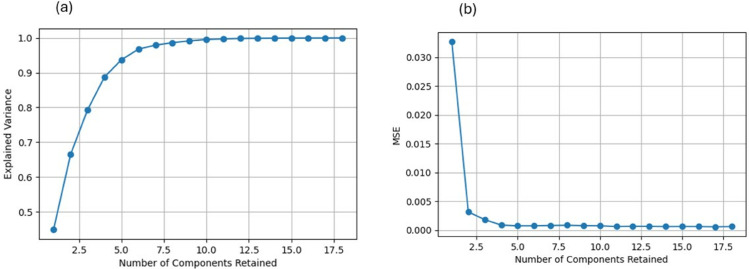
(a) Explained variance as a function of the number of components retained in the logistic regression model. (b) Mean squared error (MSE) as a function of the number of components retained in the random forest model.

To select the most informative features without impacting model performance, Principal Component Analysis (PCA) is applied in this study. PCA transforms a large set of variables into a smaller one while retaining most of the information from the original set. A standard guideline for PCA is to maintain enough principal components to explain a significant portion of the variance. In Fig 3(a), the relationship between the number of components and their corresponding explained variance is depicted, suggesting that retaining six to eight components can capture more than 95% of the variance. The regression model results recommended retaining the variables altitude (alt), isothermality (bio_03), temperature seasonality (bio_04), maximum temperature of the warmest month (bio_05), and annual precipitation (bio_12).

The number of components and the corresponding mean square error (MSE), calculated using the Random Forest model, are provided in Fig 3(b). The output suggests retaining three to six variables while achieving a significantly low MSE. The most informative features identified by the Random Forest model are altitude (alt), mean diurnal range (bio_02), and isothermality (bio_03).

**Fig 4:**
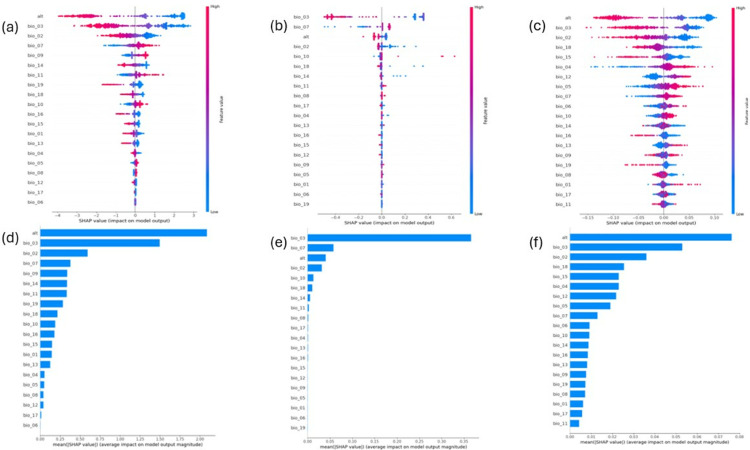
Feature importance analysis. Feature importance analysis using SHAP for three models: (a, d) logistic regression, (b, e) random forest, and (c, f) deep neural network.

The SHapley Additive exPlanations (SHAP) analysis results indicate how significant each factor contributes to the model’s prediction of its output. This is done by running a large number of predictions and comparing the impact of each variable against the other features. Fig 4(a), 4(b), and 4(c) visually present these influences on predicting the presence of the *C. pipiens* in the logistic regression model, random forest model, and deep neural network model, respectively.

In all three models demonstrated in Fig 4(d), 4(e), and 4(f), altitude (alt) and isothermality (bio_03) appear to be the most predictive variables of the presence of *C. pipiens*. Moreover, high altitude consistently decreases the likelihood of the presence of the *C. pipiens* across all models. This suggests a negative association between altitude and the presence of the *C. pipiens*.

Backward elimination was implemented during the feature selection process. Beginning with a full set of features, it iteratively removes them one by one and monitors the resulting changes in the model performance. The decision to retain a feature is determined by evaluating various model performance metrics. These metrics, including accuracy, precision, recall, and F1-score, are summarized in Table 3 for different feature combinations in the logistic regression, random forest, and DNN models. These indicators provide insights into model performance under different feature selection scenarios.

**Table 3:** Performance comparison of logistic regression, random forest, and deep neural network models.

Model	Feature	Accuracy	Precision	Recall	F1-Score
Logistic Regression	Full set of Variables	0.99	0.99	0.99	0.99
Logistic Regression	bio_03, 11, 12, 19	0.99	0.99	0.99	0.99
Logistic Regression	alt and bio_12	0.99	0.99	0.99	0.99
Random Forest	Full set of Variables	**1**	**1**	0.99	0.99
Random Forest	bio_03, 11, 12, 19	**1**	**1**	0.99	0.99
Random Forest	alt and bio_12	**1**	**1**	0.99	0.99
Deep Neural Network	Full set of Variables	**1**	**1**	0.99	**1**
Deep Neural Network	bio_03, 11, 12, 19	**1**	**1**	0.98	0.99
Deep Neural Network	alt and bio_12	**1**	**1**	**1**	**1**

After comparing the results in Table 3, we conclude that retaining the variables of altitude (alt) and annual precipitation (bio_12) from the complete set of features is the most appropriate course of action. These two variables exhibit substantial predictive power and make a significant contribution to the models’ overall performance.

The Random Forest and Deep Neural Network models outperformed Logistic Regression in terms of accuracy, as shown in Table 3. The DNN achieved the best results among the three models, mainly when using the altitude (alt) and annual precipitation (bio_12) feature set. This indicates the DNN model’s capability to extract and utilize complex patterns from the data. While logistic regression usually shows reliable and consistent performance, it is less effective in capturing nonlinear relationships than RF and DNN. These findings suggest that selecting appropriate feature sets and employing advanced models, such as RF or DNN, can substantially enhance predictive performance.

### ML, DL, and RL models performance

In this subsection, we compare the performance of machine learning (ML), deep learning (DL), and reinforcement learning (RL) methods in the context of two different sets of variables that we identified in the previous subsection. Logistic regression, RF, and DNN consistently demonstrate strong performance, achieving high accuracy, precision, recall, and F1 Scores, as shown in Table 4. The performance of the four RL methods is quite similar, with slight variations observed in precision, recall, and F1-score.

**Table 4:** Comparison of performance of machine learning, deep learning, and reinforcement learning models.

Models	Variable Set	Accuracy	Precision	Recall	F1-Score
Logistic Regression	Full set of Variables	0.99	0.99	0.99	0.99
Random Forest	Full set of Variables	**1**	**1**	0.99	0.99
Deep Neural Network	Full set of Variables	**1**	**1**	0.99	**1**
Q Learning	Full set of Variables	0.93	0.92	0.92	0.92
DQN Method	Full set of Variables	0.96	0.95	0.95	0.95
REINFORCE	Full set of Variables	0.96	0.97	0.94	**0.96**
AC Method	Full set of Variables	0.86	0.86	0.85	0.86
Logistic Regression	alt and bio_12	0.99	0.99	0.99	0.99
Random Forest	alt and bio_12	**1**	**1**	0.99	0.99
Deep Neural Network	alt and bio_12	**1**	**1**	**1**	**1**
Q Learning	alt and bio_12	0.93	0.93	0.93	0.93
DQN Method	alt and bio_12	**0.97**	**0.97**	**0.97**	0.96
REINFORCE	alt and bio_12	0.95	**0.98**	0.92	0.95
AC Method	alt and bio_12	0.82	0.79	0.86	0.82

The Q-learning method yields consistent results as the number of features decreases. This suggests that Q-learning effectively learns from both the full set of variables and the selected two variables. Similarly, the DQN and REINFORCE methods perform well in both scenarios, achieving high accuracy. In contrast, the Actor-Critic (AC) method shows slightly lower performance than the other algorithms in both scenarios. While it achieves reasonable accuracy, recall, and F1-score, it falls short in precision, particularly in the scenario with only two selected variables. Notice that the AC model trained on a broader set of bio-climatic variables tends to perform slightly better than the model trained on only two selected variables, indicating its sensitivity to feature selection.

Furthermore, DQN achieves the accuracy of 97% with the selected characteristics, compared to the highest accuracy of 99.81% by random forest, according to Table 4. Although traditional supervised models, such as Random Forest and Deep Neural Networks, outperform RL methods in terms of accuracy and precision, RL methods like DQN and REINFORCE achieve competitive performance in the case of the two selected features. This implies that RL methods perform efficiently with reduced features.

Additionally, RL methods utilize multiple random seeds to ensure generalization. This property of RL methods can potentially reduce overfitting compared to other supervised learning models, which may require regularization techniques to achieve similar performance. Additionally, RL methods can optimize for diverse objectives beyond traditional metrics, such as accuracy or F1-score. For example, they can be modified to maximize cumulative rewards, balancing precision and recall depending on the task’s priorities. Moreover, RL models can adapt to changing environments by continuously learning and improving optimal decision-making strategies. This ability makes them suitable for real-time species prediction applications.

### Predicted maps

**Fig 5:**
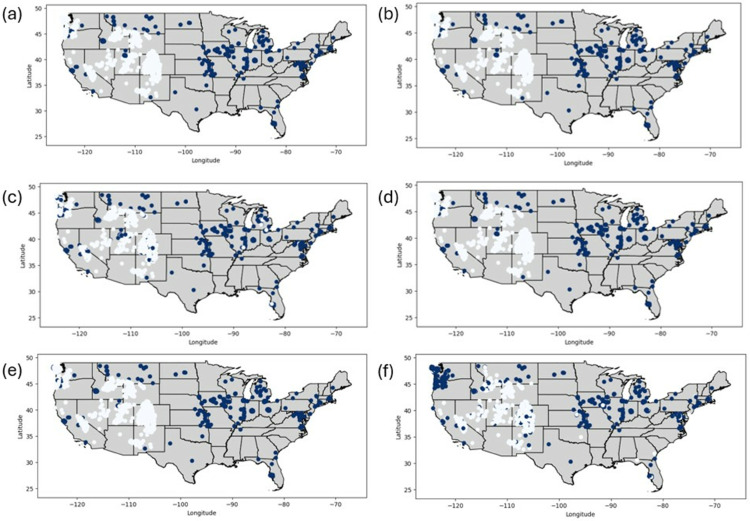
Presence and absence of *C. pipiens* predicted by different models: (a) Random Forest, (b) Deep Neural Network, (c) Q-Learning, (d) Deep Q-Network, (e) REINFORCE, and (f) Actor-Critic. Blue points indicate locations suitable for *C. pipiens*, while white points represent less suitable locations across the United States.

Predictive maps of habitat suitability for *C. pipiens* using each of the ML, DL, and RL models are shown in Fig 5. The spatial presence of regions classified as suitable (blue areas) or less appropriate (white areas). We find that all models exhibit consistent agreement in identifying key areas of suitability, such as parts of the Midwest and East Coast. Maps of random forest in Fig 5(a) and of Deep Neural Network in Fig 5(b) predict more conservative presences in the western United States. However, different predictions of suitable regions are also observed; for instance, some places in Colorado are marked as culex suitable areas based on the Q-Learning in Fig 5(c), DQN in Fig 5(d), REINFORCE in Fig 5(e), and Actor-Critic in Fig 5(f).

## Discussion

Predicting the presence of mosquitoes is important to public health due to their role as vectors of several human pathogens. Although previous studies have shown the influence of climate factors on mosquito populations, accurately modeling their presence remains a challenge. Climate change and other ecological factors affect mosquito habitats in a way that necessitates predictive modeling for public health and vector control strategies. The MaxEnt model, commonly used in ecological research, proved quite useful in modeling species distributions when absence data were unavailable. In this study, we compared multiple predictive modeling techniques to predict mosquito presence based on historical data and bioclimatic conditions. We use absences predicted by MaxEnt as the true absences in the comparative study.

To achieve this, we used and compared several machine learning methods, including Logistic Regression, RF, DNN, and RL algorithms. By comparing these methods, we assess their effectiveness in capturing the ecological complexities that affect mosquito presence. The results highlighted the strengths and limitations of each approach.

Logistic Regression provided a baseline for binary classification by identifying the presence and absence of mosquitoes based on climate variables. Random Forest showed an improvement in predictive accuracy and robustness due to its ability to handle complex feature interactions and nonlinearities. DNNs were also able to capture complex relationships within the dataset, which helps in predicting the effect of climate variations on mosquito presence. Finally, RL introduced a dynamic learning approach that enables adaptive decision-making, improving the prediction of presence and absence over time.

Our findings suggest that when presence and absence data are available, traditional machine learning models, including logistic regression, RF, and DNNs, consistently achieve high accuracy, precision, and recall. Among the four RL methods we examined here, Q-Learning, DQN, and REINFORCE exhibited efficient predictive capabilities, especially when using a reduced set of characteristics. This emphasizes the predictive ability of those RL methods in situations with few features. The Actor-Critic algorithm showed slightly lower precision and was more sensitive to feature selection. Yet, it performed better with the larger set of variables. Although the ML models outperformed the RL algorithms in accuracy, the latter were more flexible and avoided overfitting by using random seeds and constantly learning from new data. These characteristics suggest that RL-based approaches can effectively perform dynamic and real-time mosquito presence predictions.

This study examined different machine, deep, and reinforcement learning methods in detecting the presence of *C. pipiens* using key bioclimatic features. When climate conditions were used as predictors, altitude and annual precipitation were found to be the most significant features. Our findings concur with those of Allan et al. (2009), who demonstrated that rainfall can have a substantial impact on pesticide residence periods and egg deposition [35]. Rainfall can increase the number of places where mosquitoes can live, as stagnant water can provide a suitable habitat for hatching eggs and increase the mosquito population. Ragab et al. (2024) indicated that altitude is a significant bioclimatic element that impacts the distributions of *C. pipiens* [19].

Our study is limited by its reliance on historical surveillance data and the methods used for data collection. The *C. pipiens* presence data were taken from the Global Biodiversity Information Facility (GBIF), which, while comprehensive, may be subject to convenience, sampling bias, and uneven spatial distribution, let alone that it has been collected over a long period. The uneven sampling could affect the accuracy of the species distribution models, particularly in under-sampled regions. The lack of a time component also affects the prediction, as climate exhibits seasonal changes in addition to trends associated with climate change. We employed data cleaning procedures, including the removal of duplicates and erroneous points, to enhance the data set; however, these inherent biases in the data remain a limitation. Another limitation of our study is that it focuses on a single disease-vector species, which may limit the generalization of our findings. Expanding the analysis to include multiple vector species could enhance the model’s applicability and provide more comprehensive insights into the control of vector-borne diseases.

The predictive ability of RL models applied to mosquito disease vectors for WNW, as shown in our study, provides the structure for real-time vector control and public health planning. Because RL agents are inherently suited for environments that change over time, the application allows for continuous learning and model updating as new mosquito surveillance data becomes available. This makes RL-based models particularly well-suited for adaptive management control approaches, where the control measures for mosquito populations, such as fogging or larviciding, must be rapidly deployed in response to emerging WNV hotspots. By identifying geographic areas with an increasing predicted risk of C. pipiens and WNV occurrence, public health authorities can prioritize surveillance resources and vector control interventions more effectively. Furthermore, the RL-predictive results can be used to guide health policy decisions regarding resource allocation for mosquito control programs. Budget-constrained counties or states could use these outputs to optimize the spatial targeting of interventions, minimizing disease transmission risk while maximizing cost-effectiveness. Since WNV transmission risk is highly dependent on mosquito density and local environmental conditions, deploying control measures in high-probability zones identified through RL models could significantly reduce human transmission to WNV-carrying mosquitoes. Integrating RL models with real-time environmental surveillance data enables rapid response for public health. It provides an evidence base for adaptive management decision making to reduce vector-borne disease transmission, especially in identified emerging hot spot areas.

## Supporting information

S1 Appendix Additional results. Grid search results, learning curves, distribution map and confusion matrix.
(PDF)
