# Supplementary material for: Comparing machine learning, deep learning, and reinforcement learning performance in Culex pipiens predictive modeling
Source: PLoS One. 2025 Nov 13;20(11):e0333536. doi: 10.1371/journal.pone.0333536 (PMC12614784; doi:10.1371/journal.pone.0333536)
Supplement: S1 Appendix — Grid search results, learning curves, distribution map and confusion matrix. (PDF) [file pone.0333536.s001.pdf]

Appendix

1. Grid search results

Hyperparameters are not learned during the training process but are predetermined by researchers. Optimizing these hyperparameters is crucial to enhancing the model's performance. The tuning process involves experimenting with various combinations of hyperparameter values to find the combination that yields the best performance from the model. In each model, we have tried out a wide range of values, the results of grid search applied to random forest and reinforcement learning methods are provided in Tables 4.1, 4.2, 4.3, 4.4, and 4.5.

In the RF model, We try to adjust the following set of hyperparameters: the maximum number of levels in each decision tree, the maximum number of features considered for splitting a node, the minimum number of data points allowed in a leaf node, the min number of data points placed in a node before the node is split, and the number of trees in the forest.

To determine the maximum depth of the trees in the Random Forest model, see Table 4.1, values of 20, 25, and 30 were tested, and 20 is the recommended value. Additionally, Other hyperparameters were tested, including the maximum number of features for node splitting, the minimum number of samples at the leaf and internal node, and the number of decision trees in the model.

Table 4.1: Hyperparameter Tuning in Random Forest

Hyperparameters	List of values	Recommended Value
Max Depth	20, 25, 30	20
Max Features	15, 20, 25, 30	15
Mini Samples Leaf	1, 2, 3	1
Mini Samples Split	2, 3, 4	2
Number of Estimators	200, 300, 400	200

In the Q-Learning algorithm, the learning rate determines how much new information updates the Q-values. A higher learning rate makes the agent quickly adapt to new information. On the other hand, a lower learning rate leads to slower learning, but to retain more of the past knowledge. The discount factor, ranging from 0 to 1, represents the importance of future rewards. A value close to 1 means the agent favors long-term rewards, while a value close to 0 makes it focus on immediate rewards. The exploration probability controls the balance between exploration and exploitation. A higher value of the probability of exploration encourages more exploration, while a lower value tends to favor the exploitation of learned

actions.

Table 4.2 outlines the hyperparameters examined for Q-learning. The learning rate was evaluated at 0.001, 0.01, 0.05, 0.1, and 0.3, with 0.3 recommended. The recommended values of the discount factor and exploration probability are 0.8 and 0.1, respectively.

Table 4.2: Hyperparameter Tuning in Q-learning

Hyperparameters	List of values	Recommended Value
Learning Rate	0.001, 0.01, 0.05, 0.1, 0.3	0.3
Discount Factor	0.8, 0.9, 0.95	0.8
Exploration Probability	0.1, 0.2, 0.3	0.1

In a DQN model, the learning rate determines how quickly or slowly the network learns from new experiences. Batch size refers to the number of experiences sampled from the replay buffer at each training step to update the Q-network. Instead of updating after every single experience, the DQN collects multiple experiences and updates the model using a mini-batch of experiences. Batch sizes are often set to powers of 2, such as 32, 64, or 128. In this study, a batch size of 32 is chosen for the DQN model in Table 4.3, coupled with a learning rate of 0.01.

Table 4.3: Hyperparameter Tuning in DQN

Hyperparameters	List of values	Recommended Value
Learning Rate	0.001, 0.01, 0.05, 0.1	0.01
Batch Size	32, 64	32

Similarly, a discount rate of 0.9 is recommended for the REINFORCE model, as seen in Table 4.4.

Table 4.4: Hyperparameter Tuning in Reinforce

Hyperparameters	List of values	Recommended Value
Discount Factor	0.8, 0.9, 0.95, 0.98	0.9

In an Actor-Critic model, which combines an actor neural network and a critic neural network, key settings such as the actor learning rate, critic learning rate, and discount factor significantly impact how well and how quickly the model learns. The actor learning rate determines how much the actor's policy is updated in response to feedback from the critic. The critic learning rate determines how much the value function is updated when new rewards are observed. The result, as indicated in Table 4.5, recommends selecting learning rates of 0.01 and 0.05 for the actor and critic, along with a discount factor of 0.98.

2. Learning curves

Table 4.5: Hyperparameter Tuning in Actor Critic

Hyperparameters	List of values	Recommended Value
Actor Learning Rate	0.001, 0.01	0.01
Critic Learning Rate	0.005, 0.05	0.05
Discount Factor	0.8, 0.9, 0.95, 0.98	0.98

The training curve for Q-learning shows steady improvement over episodes as stated in Figure 4.1 (a), indicating effective learning with the complete set of bioclimatic variables. The reinforcement training curve demonstrates rapid learning in the few initial episodes and achieves a high reward level comparable to Q-learning. The training curves for DQN and Actor Critic exhibit fluctuations throughout the training process, indicating instability in learning. It can be observed that the DQN learning curve achieves a higher reward level than Q-learning, while the Actor-Critic reaches a reasonable reward level that is lower than Q-learning.

The testing curves generally mirror the trends observed in the training curves, indicating that the models generalize well to new data refer to Figure 4.1 (b). When trained with only two variables (alt and bio04), see Figure 4.3 (c), Q Learning maintains a stable training curve, and demonstrates a similar rate of improvement compared to the curves with all bioclimatic variables. It converges to the same reward level. Q Learning, DQN, Reinforce, and Actor-Critic trained with two variables all show similar results to the curves with all bioclimatic variables.

Similar to the training curves, the testing curves depicted in Figure 4.3 (d) for two selected variables generally show similar tendency and reward levels compared to the testing curves with all bioclimatic variables. Across all algorithms and variable sets, the testing curves tend to follow the trends observed in the training curves, indicating good generalization. However, slight performance differences may exist, especially in the case of DQN, which exhibits higher variability between training and testing. Furthermore, Actor-Critic is observed to have a larger variation in performance when trained with the full set of bioclimatic variables compared to when trained with only two selected variables.

It is noted that training with all bioclimatic variables does not exhibit faster convergence and higher reward levels compared to training with only two selected variables. This suggests that the additional variables do not significantly contribute valuable information to the learning process.

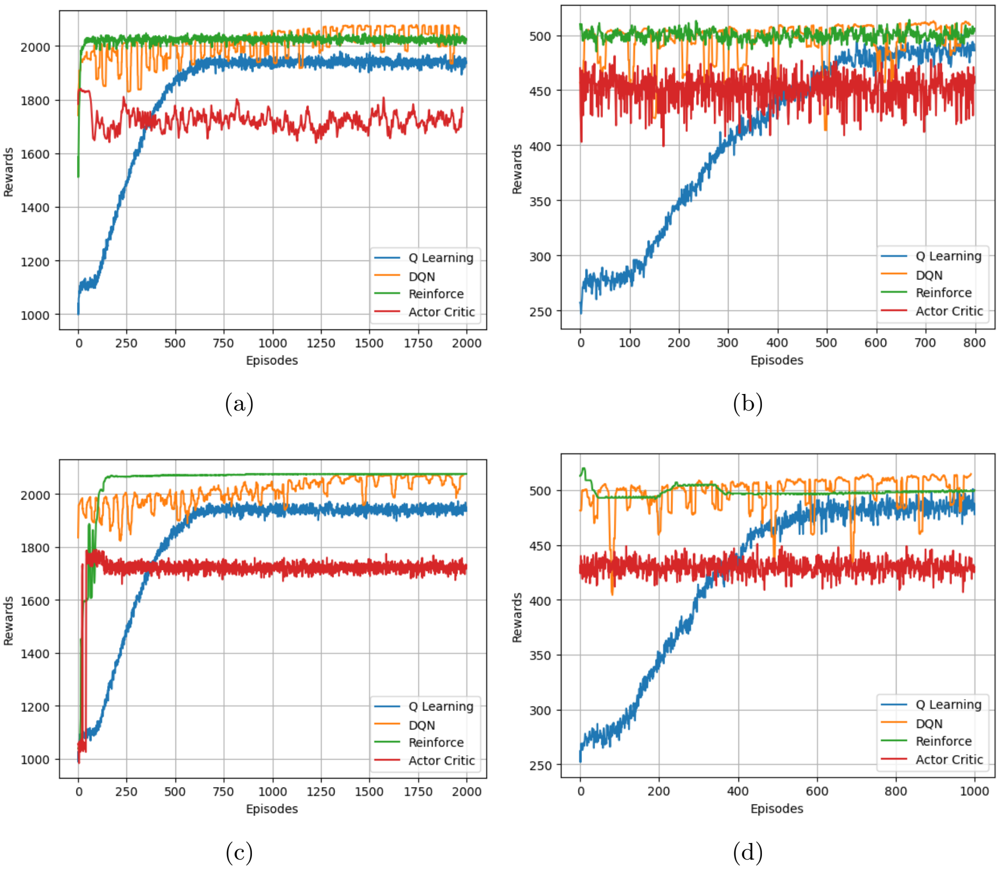

Figure 4.1: Learning curves for training and testing in RL methods. The top panel displays learning curves with all bioclimatic variables from left to right. The bottom panel shows learning curves with two selected variables from left to right.

3. Culex distribution map

In the map 4.2, the areas where *Culex pipiens* is present are shown in blue, while the gray areas indicate its absence across the contiguous United States. The presence patterns among different methods showed a high degree of similarity. The results indicate that the potential presence of *Culex pipiens* is primarily concentrated in Michigan, Indiana, Illinois, Ohio, Kentucky, Oklahoma, Missouri, Mississippi, Alabama, Virginia, North Carolina, South Carolina, Georgia, and smaller portions of Louisiana and Iowa. Additionally, the other regions that were identified as highly suitable for the potential establishment of *C. pipiens* include Pennsylvania, Maine, Vermont, New Hampshire, Massachusetts, Connecticut, Tennessee, and the southwest region of Wisconsin. Areas in Texas, central Kansas, and eastern and southern Minnesota were found to be moderately appropriate for *C. pipiens*. In contrast, North Dakota, South Dakota, New Mexico, Montana, Nevada, Utah, and northeast Alaska were deemed unfavorable locations for *C. pipiens*

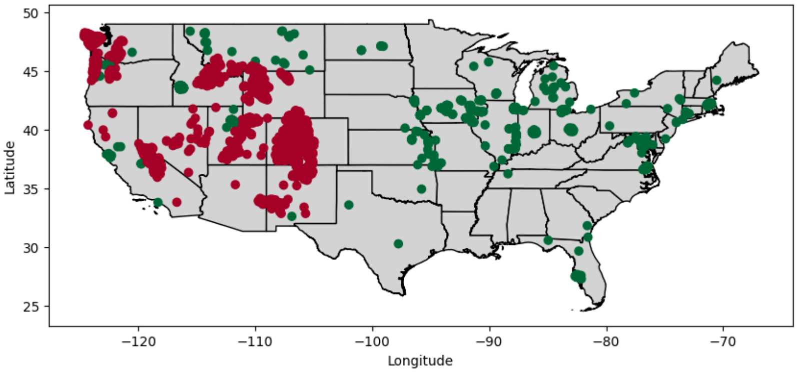

Figure 4.2: Presence of localities in USA where *Culex pipiens* has been collected or not observed. The localities where *Culex pipiens* were present (green dots) or absent (red dots) were used in the testing dataset.

in the United States.

4. Confusion matrix

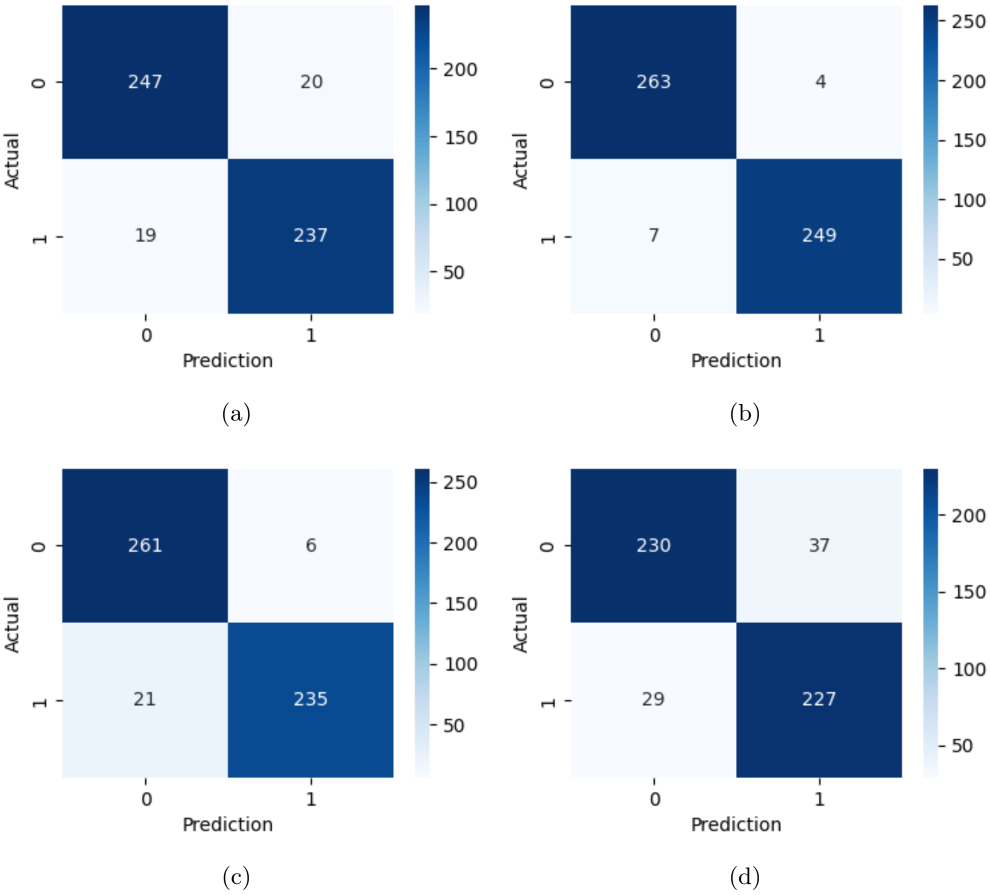

Figure 4.3: Confusion matrix for testing in RL methods. The top panel displays the Confusion matrix for Q learning and DQN from left to right. The bottom panel shows results for REINFORCE and A2C from left to right.
